# Literature Review and an Italian Hospital Experience about Post-Natal CMV Infection Acquired by Breast-Feeding in Very Low and/or Extremely Low Birth Weight Infants

**DOI:** 10.3390/nu13020660

**Published:** 2021-02-18

**Authors:** Francesca Garofoli, Elisa Civardi, Simona Zanette, Micol Angelini, Gianfranco Perotti, Marco Zecca, Giuseppina Lombardi

**Affiliations:** Neonatal and Intensive Care Unit (NICU), Fondazione IRCCS Policlinico San Matteo, Italy; e.civardi@smatteo.pv.it (E.C.); s.zanette@smatteo.pv.it (S.Z.); micol090777@gmail.com (M.A.); gf.perotti@smatteo.pv.it (G.P.); m.zecca@smatteo.pv.it (M.Z.); g.lombardi@smatteo.pv.it (G.L.)

**Keywords:** post-natally acquired cytomegalovirus, breastfeeding, preterm infants

## Abstract

Breastfeeding is recommended for all neonates due to a known variety of beneficial effects, but infants can be infected by cell-associated bacteria and viruses from breast milk, such as cytomegalovirus (CMV). The majority of CMV-seropositive breastfeeding women have a viral, self-restricted reactivation, can shed the virus in the milk for about 12 weeks after delivery, and can transmit the infection to their offspring. Post-natal CMV-infected term infants are mainly asymptomatic, while very low birth weight (VLBW, <1500 g) and extremely low birth weight (ELBW, <1000 g) infants may present with severe disease, short-term sequelae ranging from abnormalities in laboratory indexes to sepsis-like syndrome, and long-term sequelae such as developmental problems. Thus, the use of thermally treated maternal milk for VLBW/ELBW infants may be indicated to prevent/reduce the risk of CMV transmission. Different techniques, with varying efficacy in eradicating CMV and maintaining the activity of biological compounds in milk are available: long/short pasteurization, freeze-thawing, the use of microwaves, and ultraviolet-C irradiation. In our NICU, the use of maternal raw milk is always strongly recommended for term/preterm infants, but to reduce risk of CMV transmission, freeze-thawing mother’s own milk is used in neonates with GA ≤ 30 weeks or/and weight ≤ 1000 g, usually regardless of serological maternal condition, as CMV screening is not routinely offered to pregnant women and the milk of seroimmune mothers is not evaluated for CMV reactivation, as its rate is similar to seroprevalence. Over the last 4 years, we had 10 VLBW/ELBW newborns in our NICU with late-onset sepsis and negative cultures. In these cases, the research of CMV DNA in neonatal urine or saliva, for the diagnosis of post-natal symptomatic infection (once congenital transmission has been excluded) may be useful and not invasive. The take-home message we would like to share is that acquired CMV infection should be considered in VLBW/ELBW infants breastfed by seropositive mothers and presenting severe symptoms—particularly sepsis with negative cultures. This could allow pediatricians to make better-quality diagnoses, perform supportive therapy, provide antiviral treatment if needed, or establish a “pre-emptive” therapy for these high-risk neonates.

## 1. Introduction

Breastfeeding is recommended for all term and preterm infants. The beneficial effects of breast milk for preterm infants, both in extremely low birth weight (ELBW, <1000 g) and very low birth weight (VLBW, <1500 g) infants have been reported on growth and developmental outcome, also providing protection against infections, sepsis, and necrotizing enterocolitis (NEC) [1,2]. The neonates benefit from the nutritional components of breast milk and from immunological and anti-infectious bioactive factors such as immunoglobulins, antibody activators, antioxidants, cytokines, lactoferrin, oligosaccharides, and other milk compounds [1,2,3]. This combination of molecules is able to produce a positive imprint on the neonatal gut with precocious microbial colonization, and contributes to the development of the immune system, leading to a variety of different positive effects [4]. Thus, breastfeeding demonstrates beneficial effects on growth and developmental outcome, and has protective effects against infections such as sepsis and necrotizing enterocolitis (NEC) [1,2,3,4], as well as a variety of non-infectious diseases such as diabetes, malignancies (leukemia, lymphoma), obesity, and sudden infant death syndrome (SIDS). There are also maternal health benefits to breastfeeding, such as decreased postpartum bleeding, more rapid uterine involution, decreased menstrual blood loss, increased child spacing, earlier return to pre-pregnancy weight, and decreased risk of breast and ovarian cancers. It also saves time and money. Finally, it also has emotional benefits for both mother and infant [5].

Nevertheless, preterm infants have an increased intestinal permeability, or “leaky gut,” that may support the passage of cell-associated viruses and bacteria in breast milk [6]. This passage through the mucosa is facilitated by the reduced production of gastric hydrochloric acid. Moreover, the immune system is not fully developed and is characterized by enhanced tolerogenic and reduced Th1 responses [7]. Cytomegalovirus (CMV) is a ubiquitous human-specific DNA virus belonging to the Herpesviridae family, which plays a major role in immunosuppressed/transplanted patients and in congenital infected neonates. In the latter group, it is the leading cause of sensorineural hearing loss and an important cause for neurologic sequelae [8]. Besides the trans-placenta passage, seropositive women can infect the neonate through cervical-vaginal secretion during delivery and through breast milk post-natally [9]. CMV seroprevalence in women of child-bearing age ranges about from 50% to 85%, while in developing countries the seroprevalence can be even higher [8]. 

Even if many studies have been performed since the 1980s regarding acquired post-natal CMV infection via breast milk, this review mainly focuses on the latest published papers, from 2010 until now. These newer publications take into account results from earlier literature and add new findings on the basis of larger investigations and improved procedures, such as the methods used for CMV detection, from viral culture to real-time polymerase chain reaction (PCR) techniques. Through this review, we aim to find a path for further discussion in order to improve the management of post-natal CMV infection in VLBW/ELBW infants and find a shared mode to face the problem.

## 2. Mammary Reactivation in Lactating CMV Seropositive Women

One study demonstrated that up to 96% of CMV-seropositive breastfeeding women had a reactivation of CMV localized in the mammary gland during lactation, shedding the virus in the milk with the possibility of transmitting the infection to their offspring [10]. CMV acquisition via breast milk probably follows a trans-mucosal route. In term infants, the infection may be transmitted at the oropharyngeal or nasopharyngeal level, while most VLBW infants are fed by enteral nutrition, in the gastroenteric tract, implying that CMV may be transferred directly through the small bowel mucosa [11]. In preterm infants, the rates of transmission of postnatal CMV infection mainly depend on prematurity level and feeding method. The following ranges have been reported in different studies: from 5.7% to 58.6% [12], from 4% to 69% [13], and from 8% to 37% [14]. In VLBW and ELBW infants, the infection can be symptomatic with a varying incidence of from 0 to 75% of infected infants [12]. Besides the newborn’s health status, the virologic/immunologic characteristics of the milk may be associated with the risk/severity of postnatal CMV infection. CMV reactivation during lactation is a local process, restricted to the breast, with no viral DNA in maternal blood and urine and with no clinical laboratory signs or symptoms of systemic infection. The dynamic of reactivation is similar in every lactating seropositive mother, irrespective of whether she acts as a viral transmitter or non-transmitter [15]. Generally, viral reactivation during lactation is a strictly self-limited process, characterized by CMV secretion into milk from the first week post-partum with a low initial viral load which peaks at about 4–8 weeks, declines, and finishes by week 9–12 post-partum [15,16]. Regrettably, the exact time of virus secretion into milk is characterized by inter-individual variety, so only by measuring viral load or viral DNA concentration in milk is it possible to identify the risk of transmission [16]. CMV load and CMV-specific cellular and humoral immune responses of seropositive mothers of VLBW infants with/without symptomatic postnatal infection are similar, while inverse correlation has been found between CMV IgG avidity and CMV load in milk [13]. This finding suggests that an enhancement of maternal CMV-specific IgG responses could reduce CMV shedding into breast milk based on a number of papers demonstrating an association between high viral load in breast milk and risk of infection transmission. This is an important finding for possible preventive strategies [9,10,16]. Real-time PCR can detect CMV DNA concentration in breast milk, which can help to establish an approach to avoid or mitigate viral transmission [17]. Preventing infection is important because morbidity among full-term infants is minimal, but can be severe for VLBW and ELBW infants, with short- and long-term sequelae. 

## 3. Short- and Long-Term Sequelae in Preterm Infants with CMV Infection via Breast-Milk

Post-natally infected term infants are mainly asymptomatic, possibly thanks to the protection of maternal IgG passing through the placenta after 28 weeks of gestation [9]. Since preterm VLBW and ELBW infants born before this period do not have this inheritance, they can be symptomatic if the infection passes through breast milk.

### 3.1. Short-Term Sequelae

Short-term sequelae include hepatosplenomegaly, hepatitis, altered hematological parameters (neutropenia, lymphocytosis, thrombocytopenia), pneumonia, and sepsis-like syndrome (SLS) [11,18,19], followed by bronchopulmonary dysplasia (BPD) and/or necrotizing enterocolitis (NEC) [11,18,19,20,21,22].

#### 3.1.1. Rate of Infection Transmission, Symptoms: SLS

A meta-analysis estimated that the rate of infection in VLBW infants fed with untreated breast milk was 19%, and the rate of SLS was 4%. In infants fed by frozen breast milk, rates were respectively 13% and 5%—a slightly lower rate of acquired CMV infection and a similar incidence of CMV-SLS [18].

Another meta-analysis showed a range of CMV breastfeeding transmission of 5.7–58.6% with a symptomatic disease of 0–34.5% (median 3.7%) and SLS of 0–13.8% (median 0.7%) [19].

One study demonstrated that conventional pasteurization completely prevented CMV transmission compared to a significantly higher incidence (10%) when feeding >60% freeze-thawed breast milk [20].

#### 3.1.2. Bronchopulmonary Dysplasia (BPD) and/or Necrotizing Enterocolitis (NEC)

The same study [20] demonstrated that BPD (≥moderate) was significantly associated with CMV infection, while NEC, retinopathy of prematurity (ROP), intraventricular hemorrhage (IVH), and periventricular leukomalacia (PVL) were equally distributed between infected and non-infected preterm infants [20].

A matched retrospective cohort study including a large number of VLBW infants from many neonatal intensive care units (NICUs) in the United States showed that post-natal CMV infection via breast milk statistically increased risks of death and BPD [21]. A multicenter cohort study showed that postnatal CMV infection in VLBW infants correlated with an 80% relative increase in the risk of having a failed hearing screen, increased postnatal age at discharge, lower weight at 40 weeks post menstrual age (PMA) and an increased risk of BPD, but not an augmented risk of NEC [22].

A multicenter cohort study did not reveal an association between maternal milk, postnatal CMV infection, and risk of BPD, but highlighted an association with NEC (18% of infected vs. 7% of non-infected babies), the exposure to a higher viral load was associated with a twofold higher risk of NEC [23]. A case report describing the association between postnatal breast milk CMV infection and sepsis leading to NEC has been published [24].

Another study revealed no significant differences in mortality, length of hospital stay, BPD, IVH, PVL, ROP, SLS, cerebral palsy (CP), head circumference, and bodyweight growth [12].

### 3.2. Long-Term Sequelae

#### Neurodevelopmental Outcome

The children recruited for the previous study were also scored for long-term symptoms, growth neurodevelopmental outcome, and hearing function at 12 and 24 months of corrected age. The former CMV-infected group did not appear to have major adverse effects on the outcomes [12]. A controlled study performed at 4 years of age showed that children, previously VLBW with post-natal CMV infection, suffered from detrimental influence on cognitive development, even if complications such as CP were equally distributed between groups [25]. Another study found no differences between VLBW infants with post-natal CMV infection and controls with regard to neurologic, speech, language, or motor development at 2–4.5 years of age, and none of the children had sensorineural hearing loss [26]. Meanwhile, another paper showed that preterm children with acquired CMV infection had cognitive and motor function within the normal range, but with a poorer outcome and significantly lower complex cognitive abilities than uninfected controls [27]. A further study evaluated cognitive status in a large cohort of subjects at 6 years of age, showing that previously CMV-infected children tested normal, but with lower mean scores in all domains, significant only for verbal IQ. The regression analysis did not confirm the CMV impact, but highlighted significant influence of maternal education and ethnicity. No significant differences in motor development were found. The authors thus concluded that infection does not have lasting adverse effects on neurodevelopment within the first 6 years of life [28]. An article described the risk of neuropsychological impairment in adolescents born preterm (≤32 weeks gestational age (GA) or 1500 g birth weight), compared to these born at term. The previously preterm children had significantly lower scores in all the assessments, and those adolescents who were also CMV-infected scored significantly lower in overall cognitive function, but not in visual-perceptive abilities, than the non-infected preterm controls [29]. A systematic review also considering long-term outcomes revealed a low risk of mild neurological and cognitive sequelae, with no sensorineural hearing loss in preterm symptomatic infected infants [19]. A magnetic resonance imaging (MRI) study detected long-term neurobiological consequences in preterm infants with postnatal CMV. The study suggested that signs of postnatal CMV infection are detectable in older children and adolescents, compatible with a higher effort when performing a cognitive task [30]. The cited literature highlights a high inter-individual variety as regards the rate of CMV transmission, symptoms, and short- or long- term sequelae for VLBW and ELBW infants, breastfed by seropositive mothers. On this basis, it is not possible to predict with certainty to what extent post-natal CMV infection could be responsible for the described signs and symptoms. This confirms the need for further studies investigating pathologies attributable to acquired CMV infection in breastfed preterm newborns.

## 4. Prevention Strategies

The contrasting literature on post-natally acquired CMV infection by breast milk in VLBW or ELBW infants does not support a univocal strategy based on either the recommendation/avoidance of the use of raw breast milk or of any kind of treated or formula milk. As mentioned previously, breast milk is the optimal food for infants, in particular for preterm infants, including VLBW and ELBW, providing advantages such as preventing ROP, NEC, and infections, and improving neurological development [1,2,3]. In 2012, the American Academy of Pediatrics promoted breastfeeding in all newborns, at least during the first 6 months of life, in particular emphasizing the benefits of feeding human milk to high-risk VLBW premature infants. The value of routinely feeding human milk from seropositive mothers to preterm infants outweighs the risks of clinical disease, especially because no long-term neurodevelopmental abnormalities have been reported. [31]. At present, the opinion on the matter is controversial due to the possibility for VLBW and ELBW infants to develop a severe symptomatic CMV infection, and because the data on neurodevelopment are divisive, as emerging data shows. In 2018, the Red Book Committee of the American Academy of Pediatrics suggested the use of short-term pasteurization of breast milk for premature infants born at <32 weeks, depending on serological CMV maternal status [11,32]. Different protocols to inactivate CMV in breast milk have been investigated, with the target of eradicating or significantly diminishing the viral load while simultaneously preserving the nutritional and immunological components.

### 4.1. Pasteurization

Pasteurization is the most effective procedure to inactivate CMV in breast milk, as long-term pasteurization (30 min at 62.5 °C, Holder pasteurization) has been demonstrated to fully disarm viruses (including CMV) and bacteria, but significantly reduces the amount of beneficial biological compounds in milk, as reported in recent reviews [9,11,20]. Thus, short-term viral inactivation has been investigated with different heating/cooling temperatures and times, and is standardized at 62 °C for 5 s. Short-term pasteurization conserves most of the nutritional and immunologic components of milk, such as CMV-specific antibodies, enzyme activity, hormones, and growth factors [10,33,34,35]. The possible limitations of the described procedure necessitate a specific machine capable of creating a thin milk layer, heated by a stream of hot air, following a specific ramp of temperature and duration [33,34,35]. One study in particular demonstrated the efficacy of short-term pasteurization in preventing post-natal CMV transmission, comparing preterm infants (GA < 32 weeks; birth weight < 1500 g) born to CMV immunoglobulin G-(IgG)-positive mothers with historical controls [35].

### 4.2. Freeze-Thawing

Studies have been performed with freeze-thawing procedures at −20 °C with milk storage from 18 hours to 10 days. Results demonstrated that freeze-thawing is not able to fully destroy viral infectivity, especially during peak levels of virus excretion into milk, but reduces viral load depending on its concentration [16]. A meta-analysis demonstrated that in the USA, the incidence of breast-milk-acquired postnatal CMV infection in VLBW infants receiving frozen breast milk was 13% (7–24%), slightly lower than the risk among infants fed untreated breast milk, which was 19% (11–32%) [18]. A multicenter study showed an important reduction rate of 78% in the transmission of infection to the offspring of mothers using their own frozen milk [36]. While freeze-thawing was demonstrated to only partially inactivate CMV, it preserves nutritional and protective factors, including enzymatic and binding activity. As experimentally demonstrated in the late 1970s, freezing breast milk at −20 °C for up to 3 months did not alter lactoferrin, lysozyme, or IgA levels [37].

### 4.3. Microwave and Ultraviolet-C Irradiation

Other new techniques are being investigated, among which are microwave and ultraviolet-C irradiation. A study compared CMV inactivation in milk by freeze-thawing (−20 °C for 1 day and for 3 days) with microwave radiation for 30 s (low-power setting, 500 W and high-power setting, 750 W). Only the high-power microwave radiation eradicated CMV in milk samples. Nevertheless, microwave procedures have to be fully clarified regarding preserving breast milk compounds, particularly immunoglobulins [38]. One study evaluated ultraviolet-C irradiation at 254 nm on human donor milk, demonstrating that it preserved lactoferrin, lysozyme, and secretory IgA better than Holder pasteurization. CMV inactivation has been reported, but also residual CMV proteins have been found, indicating that some viral gene transcription is active [39].

### 4.4. Others

Other techniques such as CMV immunoglobulin and/or the leukofiltration of maternal milk are currently being considered, as is the use of “pre-emptive therapy” [11,24].

In the area of prevention, one interesting molecule is lactoferrin, a natural component of breast milk. Its neutralizing activity against CMV by inhibiting viral cell entry rather than immune system stimulation has been experimentally demonstrated both in vitro and in vivo [40]. A recent study demonstrated that the lactoferrin concentration in the breast milk of transmitter and non-transmitter seroimmune CMV mothers had no significant difference, and its concentration in maternal milk was not sufficiently high to be effective [41]. Further studies have to be conducted to investigate if higher concentrations of lactoferrin can be active against CMV transmission via breastfeeding.

### 4.5. Vaccine Strategy

Vaccine is also under consideration. A study performed in Uganda evaluated CMV-specific immunoglobulin G responses in mothers at delivery and their infants’ CMV status at 6 months of age, demonstrating that high levels of glycoprotein B-specific IgG may contribute to partial protection against postnatal CMV infection. These findings support the need for further studies on glycoprotein B antigens for the possible development of a CMV vaccine [42].

### 4.6. Prevention in Different Countries

In Europe, different protocols are adopted to prevent CMV infection by breast milk in preterm infants. In France, feeding raw breast milk is recommended for infants with GA > 32 weeks or >1500 g birth weight, while pasteurization for milk from CMV-seropositive mothers is strongly suggested below these limits [11,43]. In Sweden, if infants are born before 32 weeks GA from CMV-seropositive mothers, only frozen breast milk is suggested. The Austrian Society of Pediatrics recommends even more restrictive actions to control infection from seropositive mothers using Holder-pasteurized breast milk until the corrected age of 35 weeks. In Germany, for infants with birth weight < 1000 g and/or GA < 30 weeks, thermally inactivated breast milk is recommended [15,44]. One study aiming to evaluate different approaches in feeding very preterm infants administered a standard survey to the head of the Neonatal Intensive Care Units (NICUs) in 19 regions and in 11 European countries, including Italy. Results varied widely across the countries. Fifty-six percent of units reported using raw maternal milk without restrictions regarding gestational age, birth weight, or risk of CMV transmission. The authors underlined differences in NICUs in France and Italy: among these units, 47% of CMV-seropositive mothers used pasteurized human bank or their own milk, 26% used their own frozen-thawed milk, 3% their own fresh untreated milk, and 24% formula milk [14]. In Italy, different internal procedures are used in different hospitals.

As a conclusion, we can say that the most used techniques are currently pasteurization and freeze-thawing. Pasteurization is the most efficient method to eradicate the postnatal acquisition of CMV infection through breast milk, but might affect milk quality and benefits. Short-term pasteurization may be an alternative that better conserves biological components. Freeze-thawing is used because it preserves the bioactive compounds of the maternal milk and decreases the CMV transmission rate, but it does not completely prevent the transmission of CMV. Thus, further investigations are required to better understand the advantages and risks of any techniques, and particularly to clarify the potential of novel procedures.

## 5. Monitoring VLBW Newborns with Breast-Milk-Acquired CMV Infection

Recent literature has proposed that a routine monitoring of CMV DNA levels in VLBW preterm infants with breast-milk-acquired infection could predict infants who may need intervention, avoiding or mitigating the possible onset of severe symptoms [24,45]. A case report described an infant who acquired CMV from frozen breast milk by gavage, presenting increasing CMV DNAemia long before developing CMV syndrome and NEC. Ganciclovir (GCV) therapy was thus started at 6 mg/kg/dose intravenously (IV) every 12 hours for 3 weeks. After the 3-week course of therapy, clinical signs of improvement and resolution of DNAemia confirmed the treatment success [24]. After ruling out the congenital infection within 3 weeks of life, regular screening for early detection of CMV DNA could be tested using PCR in infants GA < 32 weeks breastfed by CMV seroimmune mothers. CMV DNA can be quantified in blood, urine, and saliva. Salivary PCR testing is a useful and non-invasive tool for the surveillance of neonatal patients [45,46]. Different authors suggest a saliva-based PCR assay, performed on a weekly basis, for VLBW infants at high risk of developing severe CMV related disease via breast milk. Screening by means of salivary samples to detect CMV DNA has been shown to be highly sensitive and suitable for the parents [11,45,46]. However, this kind of screening has not been fully assessed, and further studies are required to support this as a univocal routine procedure.

## 6. Therapy

Apart from few case reports and ongoing research, there are no clinical trials suggesting the use of GCV or valganciclovir (V-GCV) in CMV infection acquired postnatally through breast-feeding [15,24,47,48]. Intravenous (IV) GCV and its oral prodrug V-GCV are the suggested therapies for congenital CMV affected infants presenting moderate to severe symptoms [49], or with evidence of central nervous system (CNS) disease [50]. Thus, the same antiviral therapy might be useful when a VLBW breast-milk-infected infant starts to present CMV-related severe signs and symptoms. Therapy for high-risk infants can be approached with the dosage for infants >32 weeks GA of GCV 6 mg/kg twice a day or V-GCV 16 mg/kg twice a day orally, monitoring the weight increase, the symptoms of CMV diseases, and possible side effects. In spite of many adverse events (AEs) reported by the use of GCV/V-GCV in neonates, children, and adults, including congenital infection or infection consequent to immunosuppression due to transplant procedures, literature regarding post-natal infection by breastfeeding describes only one case of increased transaminases and cholestasis due to GCV treatment in a preterm patient [15]. In another paper, the authors clearly declared that any AEs were attributed to GCV/V-GCV therapy in four preterm infants treated for post-natal infections. Full blood count and liver enzymes were checked weekly and found to be normal [48]. Particular attention has to be given to the duration of therapy. In the described case reports, the course lasted for a maximum of 3 weeks, with the exception of one patient, treated for 6 weeks [48]. Recently, antiviral treatment for 2 weeks has been suggested, with the CMV viral load tested weekly. The authors suggest that if symptoms persist and CMV viral load is not suppressed, treatment may continue for a further 2-week block. Since resistance to CMV has been reported in the literature at least after 8 weeks of therapy, treatments lasting more than 8 weeks are not recommended [45]. Some authors proposed monitoring CMV DNA in VLBW preterm infants during the breastfeeding period, in order to consider a “pre-emptive” treatment when the viral load increases, as happens for the prevention of severe symptoms and CMV end-organ disease in immunocompromised patients [11,24].

## 7. Local Experience

In our hospital, we have about 2000 births per year and breast-feeding is strongly promoted by clinical staff. When maternal milk is not accessible, formula milk is chosen because we do not have a local donor human milk bank. On the other hand, if maternal milk is available and enteral feeding is possible, in neonates with GA ≤ 30 weeks and/or birth weight ≤ 1000 g who are at risk of breast-milk-acquired symptomatic CMV infection, the neonatologists in our NICU follow the procedure of freeze-thawing regardless of maternal serological condition, as CMV screening is not routinely offered to pregnant women. Moreover, the milk of seroimmune mothers is not evaluated for CMV reactivation, its rate being similar to seroprevalence. Thus, maternal milk is frozen at −20 °C then stored for a minimum 72 h, and preferably for 96 h. The use of maternal raw milk is usually suggested if maternal serology is undoubtedly negative for CMV and after 30 weeks GA. The freeze-thawing procedure is affordable and effective in containing the viral load, thus mitigating the rate of CMV infection and keeping the biological/immunological compounds of the raw milk active and functional, but not eliminating the risk of transmission [37,51,52]. Usually fresh colostrum is recommended for preterm neonates, considering the low viral load at the beginning of lactation, since the beneficial effect of un-treated maternal milk is preferred when weighed against the loss of active biological compounds in thermally treated milk. After 12 weeks of life, the freezing procedure is usually reconsidered because viral shedding through the mammary gland is significantly reduced or run out, starting from week 9 to 12 post-partum. In fact, the average time to post-natal infection has been reported to be close to the DNAlactia or virolactia peak, which ranges between 4 and 8 weeks after delivery [15,16,52]. Consequences of post-natal CMV infection involve a minority of VLBW/ELBW infants, so physicians of the NICU prefer to follow a single detailed decision for each preterm infant regarding the timing of feeding with raw or treated maternal milk, mainly based on the clinical status of the preterm infant. The empirical approach to breast-fed preterms at risk of developing a CMV-related severe disease is due to the fact that the milk of seroimmune mothers is not sampled to evaluate CMV breast reactivation, and offspring are rarely monitored for CMV DNA in body fluids.

Data from the last 4 years have indicated that, in our ward, we had 10 newborns (7 <1000 g and 3 <1200 g) suffering from late-onset sepsis with no microbiological confirmation. Among those with birth weight < 1000 g, three also developed BPD. When the pathogen responsible for sepsis is not isolated, but other symptoms and signs clearly indicate a sepsis disease, the latter is identified as “clinical sepsis”. This definition is a matter of debate because a negative culture needs to distinguish between real sepsis and sepsis-like/clinical sepsis, such as noninfectious or viral conditions [53].

This review of the recent literature made us re-think breastfeeding VLBW/ELBW infants from CMV seroimmune women. In these very preterm newborns with clinical sepsis, assay for CMV DNA in urine or saliva samples could be appropriate to evaluate the presence of post-natally acquired infection (after excluding congenital transmission). In particular, if the sepsis symptoms with negative cultures appear in range of the possible peak of virolactia/DNAlactia, it could be interesting and useful to verify if the neonate has CMV DNA in its body fluid, thus indicating an ongoing viral infection. This finding could provide a further option to appropriately treat the infection. Thus, as universal neonatal CMV screening at birth is not performed, excluding a congenital infection with previously stored samples, ad hoc measurements of the neonatal CMV viral load could lead us to consider GCV or V-GCV therapy.

The take-home message we would like to share is that acquired CMV infection should be considered in VLBW/ELBW infants breastfed by seropositive mothers and presenting severe symptoms, particularly sepsis with negative cultures. As reported above, this could allow pediatricians to make a better-quality diagnosis, perform supportive therapy, provide antiviral treatment if needed, or establish a “pre-emptive” therapy for these high-risk neonates.

## Data Availability

No new data were created or analyzed in this study. Data sharing is not applicable to this article.
